# Clinical Depression in the Last Year in Life in Persons Dying from Non-Cancer Conditions—Real World Data

**DOI:** 10.3390/diseases14010009

**Published:** 2025-12-28

**Authors:** Peter Strang, Anette Alvariza, Torbjörn Schultz, Linda Björkhem-Bergman

**Affiliations:** 1 Department of Oncology-Pathology, Karolinska Institutet, SE 171 77 Stockholm, Sweden; 2 Palliative Medicine and R&D Unit, Stockholms Sjukhem, SE 112 19 Stockholm, Sweden; anette.alvariza@mchs.se (A.A.); torbjornschultz@gmail.com (T.S.); linda.bjorkhem-bergman@ki.se (L.B.-B.); 3 Department of Health Care Sciences, Marie Cederschiöld University, P.O. BOX 11189, SE 100 61 Stockholm, Sweden; 4 Division of Clinical Geriatrics, Department of Neurobiology, Care Sciences and Society (NVS), Karolinska Institutet, SE 141 83 Huddinge, Sweden

**Keywords:** depression, sex-differences, palliative care, Parkinson’s disease, chronic obstructive pulmonary disease, healthcare consumption

## Abstract

Background/Objectives: Published prevalences of depression are mainly based on measurements of depressive symptoms, whereas data on clinical depressions are lacking. Our aim was to map the prevalence of ICD-10 diagnoses of depression made by physicians in routine healthcare, during the last year of life in non-cancer conditions and to study associations with clinical variables. Methods: A registry study on all persons in ordinary accommodation, dying in 2015–2023 in non-cancer conditions. Results: Of 62,228 persons dying from non-cancer conditions, 4391 (7.1%) were formally diagnosed with depression during the last year in life. Depression was significantly more common in women than in men, 8.0% vs. 6.3% (*p* < 0.001); adjusted odds ratio (aOR) 1.46 (95%CI 1.37–1.55). Prevalence of depression was highest in persons 18–44 years (18.3%) and lowest in persons >85 years old (5.7%) (*p* < 0.001); aOR 4.12 (95%CI 3.66–4.63). It was also more common in persons living in more affluent areas, aOR 1.19 (95%CI 1.10–1.29). The condition was most frequent in persons with Parkinson’s disease (9.4%) and COPD (8.2%). Depression was associated with more emergency room visits, 89.5% vs. 81.3% (*p* < 0.001), and visits in psychiatric services in the last year in life, 41.4% vs. 8.8% (*p* < 0.001). Depression was less prevalent in persons admitted to palliative care (*p* = 0.007). Conclusions: The highest frequencies were found in women, younger persons, and those living in affluent areas, but also in certain diagnoses such as Parkinson’s disease and COPD. Clinical depression in the last year of life is associated with more emergency room visits and utilization of psychiatric services.

## 1. Introduction

Persons suffering from a life-threatening illness with short lifetime expectancy often have symptoms of anxiety, insomnia, grief, and sadness [1,2]. These symptoms are normal responses to the life crisis that accompanies a life-limiting condition. In contrast, depression is characterized by a more severe and constant condition. A diagnosis of major depression is made by a physician based on formal psychiatric criteria. However, some of these criteria are normal and expected symptoms in end-of-life patients, such as “loss of energy”, “thoughts about death”, and “insomnia”, and thus it might be difficult to assess what is a normal reaction and what is a true depression [1,3,4].

A recent, large registry study of over 27,000 people with advanced cancer in the last year of life found that clinical depression (ICD codes F32-34) was diagnosed in 4.3% of the patients, more often in women than in men with 4.8% and 3.8% (*p* = 0.002), respectively [5]. Moreover, depression was most common in hematological malignancies (5.1%), followed by brain tumors (4.9%) and lung cancer (4.4%). However, similar data in non-cancer conditions based on larger studies are mainly lacking.

In the general population (not limited to end-of-life (EOL) situations), the incidence of depression is higher in women than in men [6,7]. Age also seems to be associated with the risk of depression. According to two Swedish studies, the incidence in the general population peaks in mid-life, before 45 years of age [8,9]. Moreover, low socioeconomic status has also been associated with depression, although the associations are complex [10]. 

However, the relation between these risk factors and depression in EOL patients has not been thoroughly studied as regards non-cancer diagnoses. Several life-limiting non-cancer conditions, including Parkinson’s disease, heart failure, chronic obstructive pulmonary disease, and ALS, are receiving increased attention within Swedish palliative care [11,12,13,14]. While smaller cross-sectional studies using assessment instruments have explored different depressive symptoms or “depressive mood”, there remains limited knowledge about the prevalence of clinically diagnosed major depressive disorder among these patient groups, as determined by physicians in routine healthcare. In fact, in a PubMed search, when combining depression with palliative care, hospice, or EOL, several hundred articles were found, most of them related to cancer. When limiting the search to major non-cancer diagnoses such as the four mentioned above, we only found some occasional smaller studies. Thus, there is a knowledge gap, as one cannot generalize findings regarding chronic diseases in midlife, to the last years of life when people begin to realize that their lives are coming to an end.

Thus, the aim of this study was to map the prevalence of ICD-10 diagnoses of depression in routine health care during the last year of life in persons suffering from non-cancer diagnoses. An additional aim was to examine associations between depression and factors such as sex, age, specific non-cancer diagnoses, socioeconomic status, and health care utilization.

## 2. Materials and Methods

The Strengthening the Reporting of Observational Studies in Epidemiology (STROBE) criteria were used [15].

### 2.1. Study Design

This study was conducted on retrospective data from Region Stockholm’s administrative database, which is used for the follow-up of all forms of outpatient and inpatient healthcare utilization within Stockholm County, covering almost 2.5 million inhabitants.

### 2.2. Population

Persons aged over 18 years in ordinary accommodation with a non-cancer diagnosis who died between 2015 and 2023 were included. Those with advanced cancer disease, as well as persons living in nursing homes were excluded.

### 2.3. Variables

All data used in this study were retrieved from the administrative database within Stockholm County. Depression was identified using the clinical International Classification of Diseases 10th Revision (ICD-10) diagnoses that have been set by physicians in connection with regular health care visits in outpatient or inpatient settings. The following ICD-10 codes were included: F32, F33, and F34.

Other variables used to characterize persons with depression were as follows: age groups, sex, primary diagnosis during the last 3 months of life, socioeconomic status on area level as measured by the Mosaic system, and admission to specialized palliative care (yes/no) during the last year in life. In addition, data on place of death were collected.

Mosaic is a measure used to identify limited residential areas with similar socioeconomic characteristics, described in detail elsewhere [16]. Mosaic group 1 covers the most affluent areas, whereas Mosaic group 3 is typical for less affluent neighborhoods.

Emergency room (ER) visits, visits in psychiatric services, as well as emergency hospitals as places of death were used as outcome measures in the analysis of health care consumption.

### 2.4. Definition of Main Diagnosis

Among specific diagnoses, we chose to include four major non-cancer diagnoses (heart failure, COPD, dementia, and stroke), but also ALS, which is a classical palliative care diagnosis, and PD since the risk of depression is well known in these patients.

We did not have access to the death certificate stating the cause of death. Instead, we identified the primary diagnosis in the registry the last 3 months before death. If there were different primary diagnoses listed during the last 3 months before death, they were ranked as follows: In cases of both heart failure and COPD, COPD was defined as the main diagnosis since many patients with COPD develop a secondary heart failure due to the COPD. If dementia was one of the primary diagnoses, it was chosen as the main diagnosis as persons with dementia differ in many aspects from other patients. If Parkinson’s disease (PD) was one of the main diagnoses (and dementia was not present), PD was selected. If ALS was one of the primary diagnoses (and neither dementia nor PD was not present), ALS was chosen. If stroke was present (in the absence of dementia, PD, or ALS), stroke was chosen.

### 2.5. Selection Bias and Dropouts

Since all health care interventions must be reported to the regional database as a basis for the financial reimbursement to the health care unit, the data are almost complete, except for possible errors in the entries in the registry (the human factor).

### 2.6. Study Size

No power calculations were performed as the total cohort (all deaths of non-cancer conditions) between 2015 and 2023 in Stockholm County was included.

### 2.7. Statistical Methods and Missing Data

Descriptive data was summarized as amount (*n*) and percentage or mean and standard deviation when applicable, for each variable. Comparisons between groups were performed using t-tests, Wilcoxon Rank Sum tests, and chi-square tests when applicable.

Univariable logistic regression analyses were followed by multivariable logistic regression with adjustments for sex, age group, and socioeconomic factors. The c-statistic was chosen as a measure of goodness-of-fit for multiple logistic regression models. The Hosmer and Lemeshow goodness of fit test was also applied and it confirms the model is acceptable if the value exceeds 0.05.

The SAS 9.4/Enterprise guide 8.2 was used for the statistical analysis.

## 3. Results

### 3.1. Demographic and Clinical Data

In total, 62,228 persons died of a non-cancer condition in ordinary accommodation during the years 2015 to 2023, 27,473 (44%) women and 34,755 (56%) men (the imbalance is due to the fact that older women often live in nursing homes and those persons were excluded). The baseline demographic of the persons included in the analysis is presented in Table 1. The mean age was 76.9 years, and the most common main diagnosis was heart failure, followed by COPD and stroke. Of the 62,228 persons, 16.9% were admitted to a specialized palliative care unit (specialized palliative home care, or in-patient care).

### 3.2. Prevalence of Depression

**Figure 1:**
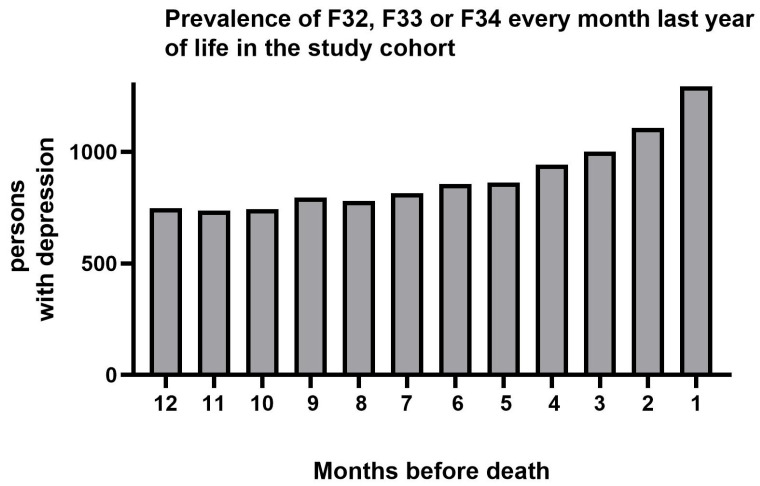
A total of 10,678 depression diagnoses were recorded across 4391 patients, detailing the monthly distribution of these cases.

Of all 62,228 persons in the cohort, 4391 (7.1%) were diagnosed with depression at least once during the last year in life, significantly more often in women than in men, 8.0% vs. 6.3% (*p* < 0.001) (Table 2). Figure 1 illustrates the distribution of diagnoses by month, with a slight increase towards the end of the last year of life. Since the same patient may have a depression diagnosis recorded more than once, the total number is higher in this figure (a total of 10,678 diagnoses during the year, distributed across the 4391 patients).

Those with depression were significantly younger, 71.6 years, vs. 77.3 years (*p* < 0.001), and the prevalence of depression varied significantly between age-groups (*p* < 0.001) and was highest in the youngest age-group, 18–44 years (18.3%) and lowest in the age group ≥85 years old (5.7%) (Table 2). The condition was most frequently found in persons with PD (9.4%) followed by COPD (8.2%) and ALS (7.0%). The frequency was higher in persons living in more affluent neighborhoods compared to those living in less affluent areas (*p* = 0.005). Persons with depression were more seldom admitted to specialized palliative care (*p* = 0.007) and had fewer hospital deaths (*p* < 0.001) (Table 2).

### 3.3. Health Care Consumption

Depression was associated with a higher incidence of emergency room visits during their last year in life, 89.5% compared to 81.3% in the persons with no depression (<0.001) (Table 3). However, persons with no depression had more emergency room visits when only studying the last month before death (*p* = 0.006). Visits in psychiatric services were also more common in persons with depression compared to those without depression, 41.4% versus 8.8% (*p* < 0.001).

### 3.4. Regression Analyses

In univariable analyses, lower age, female sex, and living in more affluent areas were significantly associated with higher odds ratios (OR) for depression (Table 4). In the multivariable analysis, the ORs for these variables were even more pronounced.

Depression was significantly more common in women than in men, adjusted odds ratio (aOR) 1.46 (95%CI 1.37–1.55), highly prevalent in persons 18–44 years; aOR 4.12 (95%CI 3.66–4.63) and more prevalent in persons living in more affluent areas, aOR 1.19 (95%CI 1.10–1.29). The c-statistic for the multiple regression model was 0.61, which implies a weak but statistically significant model.

### 3.5. Sensitivity Analysis

A sensitivity analysis was conducted to support the data from the above multivariable model (Table 4, right columns). Thus, we reran the multivariable model in three different ways: (a) by excluding the ICD-10 diagnosis F32 (Depressive episode); (b) by excluding F33 (Major depressive disorder, recurrent), and (c) by including also ICD-10 verified depressions among nursing home residents (*n* = 2853 individual residents), i.e., the group that was excluded from all other analyses. The findings are presented in Table 5, which includes the three separate models A, B, and C.

The analyses demonstrated reasonable consistency. Even after excluding ICD-10 diagnoses F32 or F33 from the analysis, age, sex, and socioeconomic Mosaic groups continued to be significant variables, though their individual effects varied. The most significant differences appeared when F32 (Depressive episode) allowing the focus to shift entirely to F33 (Major depressive disorder, recurrent). In model A, the adjusted odds ratios (aOR) for younger age groups were especially higher.

In the third comparison, data from those in ordinary housing (*n* = 4391) were merged with data concerning nursing home residents (*n* = 2853), resulting in a cohort of 7244 patients with verified depression. All statistical differences in the multivariable model (Table 4) persisted after including nursing home residents in the expanded model.

## 4. Discussion

This is, to our knowledge, the largest cohort study on the prevalence of clinical depression, based on real-world data in end-of-life patients dying from non-cancer conditions. It comprised 62,228 persons in ordinary accommodation, with 4391 cases of clinical depression. The prevalence of depression, defined by ICD-codes, in the last year of life in this population was 7.1%. Depression was significantly more common in women than in men and diagnosed most rarely in the oldest age group, ≥85 years old. Interestingly, persons living in more affluent areas had more diagnoses of depression than those living in less affluent neighborhoods. The diagnoses associated with the highest prevalence of depression were PD and COPD. Depression was associated with higher incidence of emergency room visits during the last year and significantly more visits in psychiatric services.

Although depression in the end of life has often been studied in the context of cancer, interestingly enough, depression appears more prevalent in persons dying from non-cancer conditions compared to those dying from cancer, 7.1% compared to 4.3% according to a recent study from the same part of Sweden, with the same methodology [5]. Still, the prevalence is lower compared to a previous study on depression in palliative care, who reported prevalence of 16.5% using the same ICD-codes [2]. However, they reported a heterogeneity I^2^ value of 86.8%, indicating that their data should be interpreted with great caution [2]. Since most studies on depression in the late palliative stage focus on cancer, there is reason to conduct comparative, in-depth investigations where cancer and other diagnoses are compared.

### 4.1. Clinical Variables

The incidence of depression is known to be more common in women than in men in the general populations [6,8,9,17] as well as in end-of-life patients dying from cancer [5]. In our cohort, there were fewer women because we excluded people who died in nursing homes, where the proportion of women is significantly higher than for men [18]. Nursing homes were excluded because they are run by municipalities and have more variable access to physicians.

Nevertheless, in a recent genome-wide meta-analysis it was also shown that women in general had a higher genetic risk burden for depression [7]. However, previous studies from palliative care settings have indicated that this sex-difference in incidence might be less pronounced in end-of life care [17], which is in line with our results. Indeed, young age seemed to be a much stronger risk factor than sex, according to the adjusted analysis, in good agreement with a large Danish population study on a general population [19] and also in line with Swedish data [20]. Experiencing a life-limiting condition may be particularly burdensome for younger individuals than when you are older.

The lower prevalence rates in men and in the elderly are surprising, given that suicide is most common among men in most countries, including Sweden [21,22]. In addition, in Sweden suicide is most frequent in males over 85 years of age [22,23] indicating that depression might be underdiagnosed in this age-group and among males.

In the general population, low socioeconomic status has been shown to be risk factor for depression in most countries [10] which is also in line with some studies from Sweden [9]. Interestingly, in this study population, depression was most common in persons living in more affluent areas. We do not know the reason, but this may indicate that a good socioeconomic condition is not a protective factor for depression during the last year in life. Alternatively, people with higher socioeconomic status articulate their symptoms more clearly and are therefore diagnosed more often. Moreover, the measure used for socioeconomic status is crucial: in the comprehensive review by Jacobsen et al. [10], *individual* income was strongly associated with depression, whereas the results on a *group* level were different: a sensitivity analysis showed no differences between high- and low economy subgroups, in line with the data on our Mosaic groups. Since clinical depression is likely underdiagnosed and the issue is clinically important, a qualitative study focusing on patients’ motivations and physicians’ awareness could provide a deeper understanding.

### 4.2. Specific Diagnoses

Depression was most common in persons with PD. It is well known that diagnosis of depression is frequent in PD and associated with worse quality of life, disability, and prognosis [24]. However, studies report a wide variation in prevalence rate for depression ranging from 2.7–90%, when also data on less specific depressive symptoms are included [25]. A meta-analysis showed that clinically significant symptoms of depression were present in 35% of patients with PD [26]. Recently it has also been found that persons with PD and depression experience more loneliness, and that advancing PD stage is associated with worse loneliness [27]. That study revealed that depression was one of the key predictive variables for loneliness [27].

We found diagnosed depression in 8.2% of persons dying with severe COPD, which is in good agreement with data by Wang et al. who reported 8% when using Mini-International Neuropsychiatric Interview (MINI) to identify depression, meeting the DSM-5 criteria [28]. Other studies report much higher frequencies, ranging from 10 to 65% [29,30,31], but again, those studies have used screening questionnaires, which are designed to identify psychiatric symptoms, but not to establish a diagnosis of major depression. None the less, an association between COPD and elevated levels of depression is shown in many studies [32,33]. In a recent review, depression in persons with COPD is associated with lower quality of life, functional impairment, poorer adherence to treatment, and suicidal ideation [34]. An interesting question is whether physical illness increases the risk of depression, or whether there are common factors for both conditions. Some data suggest that the same underlying genetic and environmental factors may give rise to both the underlying illness and to depression [30].

With regards to the other specified diagnoses of this study (ALS, heart failure, stroke, and dementia), some findings were a bit surprising. ALS is a diagnosis that the public fears; still, the one-year prevalence of depression of 7.0% was in line with the prevalence for the total cohort (7.1%). Again, it is difficult to compare our data with international studies, as almost all have used symptom rating scales, not clinical physician assessments. A review of such ALS studies found that 8% were assessed as having severe depression [35]. We found 5.9% depression in heart failure, which should be compared with figures around 20% in review studies [36], but these also only report data based on rating scales, rather than on clinical diagnoses. When it comes to stroke, it is even more difficult to find reliable figures as most studies report frequencies of depressive symptoms only, rather than depression. Moreover, most studies focus on long-term survivors. However, about one third seem to have some degree of depressive post-stroke symptoms [37]. Finally, with regards to dementia, the frequencies reported in the literature are much higher than ours [38], but are again based on screening tools, rather than on assessments made by physicians in everyday health care. Notably, people aged 75 and older with depression that includes dementia-like symptoms, such as forgetfulness, face a higher risk of developing dementia in the near future [39]. As for PD, associations have been found between dementia, depression, and loneliness [40]. Likewise, physical and functional health problems play a significant role in depression symptoms and increased loneliness [40].

### 4.3. Healthcare Utilization

Depression was less prevalent in persons admitted to specialized palliative care, but our study was not designed to establish causality; therefore, we cannot determine whether admission to specialized palliative care leads to fewer cases of depression, or whether individuals with less severe depression are more frequently admitted. However, several previous studies have shown that early access to palliative care could increase quality of life (QoL) in persons with advanced cancer and lead to better well-being in different areas [41,42,43,44], although outcomes are more positive in persons with cancer than those with non-cancer conditions [42].

Depression in the last year in life was also associated with more health care utilization. In persons diagnosed with depression, 41.4% needed at least one visit to psychiatric care and had a higher incidence of emergency visits. This highlights the need for cooperation between palliative care and psychiatric services, in order to provide good quality of care in persons suffering from depression in end-of-life situations. In addition, better care of persons with life-limiting conditions and diagnosed depression might decrease health care consumption.

### 4.4. Strengths and Limitations

This study provides real-world data based on a large cohort comprising 62,228 persons, without dropouts as reporting to the database is mandatory for each service to receive economic compensation. In addition, the use of the ICD-10 diagnosis made by physicians in routine health care makes the definition of depression solid. In previous reports on depression in palliative care situations, the definitions of depression used in different studies vary, often based on different assessment tools with different cut-off values and not according to diagnostic systems [4].

Register-based studies are designed to detect associations, not causal relationships, which is a limitation. Moreover, the strict use of ICD-codes might also underestimate the true prevalence of depression. Mild to moderate depressive symptoms are common in end-of-life and might not always be registered as depression in medical journals. The detection of mild symptoms is also of clinical importance, as these symptoms affect quality of life and it is often possible to alleviate such symptoms with supportive non-pharmacological measures. However, identifying major depression is particularly important, as it is this subgroup of patients with depressive symptoms who are thought to benefit most from antidepressant medication. Thus, assessments targeting individual depressive symptoms and clinical diagnostic evaluations of depression serve to complement one another.

We excluded persons living in nursing homes, as the availability of physicians at nursing homes is very limited, which possibly leads to depressions not being formally diagnosed. This exclusion might affect the generalizability of gender-specific and age-related findings, although these findings remained significant in a sensitivity analysis.

From a clinical perspective, it would have been valuable to correlate the clinical depression diagnoses with the use of antidepressant and anxiolytic medications. Unfortunately, a comprehensive ATC register for this purpose is lacking in our databases, which is a limitation.

### 4.5. Future Research

Future research should focus on how assessments of depressive symptoms and formal ICD-10-based depression diagnoses overlap. Furthermore, it would be of interest to investigate the use of antidepressant medications as well as benzodiazepines in each group, in order to assess whether there is overuse or underuse.

## 5. Conclusions

To conclude, depression is a serious problem in persons dying from a non-cancer condition and strongly associated with women, younger people, and living in more affluent areas. Depression in the last year in life is also associated with more health care utilization, especially with regards to psychiatric care. A greater awareness of the significance of depression for individuals at the end of life can lead to better support and, consequently, improved quality of life.

## Figures and Tables

**Table 1:** Baseline demographic of 62,228 persons that had died in a non-cancer condition in Stockholm County during 2015–2023. Amount in each group (*n*) and column percentage in parenthesis.

Variables	Total *n* = 62,228	Men *n* = 34,755	Women *n* = 27,473
Age, years, mean (SD)	76.9 (14.9)	74.7 (15.3)	79.8 (13.9)
Age groups			
18–44 years, *n* (%)	2564 (4.1)	1779 (5.1)	785 (2.9)
45–64 years, *n* (%)	7851 (12.6)	5370 (15.5)	2481 (9.0)
65–74 years, *n* (%)	11,565 (18.6)	7191 (20.7)	4374 (15.9)
75–84 years, *n* (%)	18,264 (29.3)	10,460 (30.1)	7804 (28.4)
85 years or more, *n* (%)	21,984 (35.3)	9955 (28.6)	12,029 (43.8)
Diagnosis			
Heart failure	8330 (13.4)	4542 (13.1)	3788 (13.8)
COPD	4466 (7.2)	2014 (5.8)	2452 (8.9)
Stroke	3859 (6.2)	1861 (5.4)	1998 (7.3)
Dementia	2172 (3.5)	1058 (3.0)	1114 (4.1)
Parkinson’s disease	593 (1.0)	391 (1.1)	202 (0.7)
ALS	501 (0.8)	279 (0.8)	222 (0.8)
Other diagnosis	42,307 (68.0)	24,610 (70.8)	17,697 (64.4)
Mosaic-Socioeconomics			
Group 1, *n* (%)	14,615 (23.5)	8144 (23.4)	6471 (23.6)
Group 2, *n* (%)	23,735 (38.1)	13,647 (39.3)	10,088 (36.7)
Group 3, *n* (%)	23,878 (38.4)	12,964 (37.3)	10,914 (39.7)
SPC			
Yes	10,522 (16.9)	5179 (14.9)	5343 (19.5)
No	51,706 (83.1)	29,576 (85.1)	22,130 (80.5)

Abbreviations: COPD = chronic obstructive pulmonary disease; ALS = amyotrophic lateral sclerosis. SPC = Admission to specialized palliative care during the last year in life. Mosaic group 1 = more affluent areas, Mosaic group 2 = middle-class areas, Mosaic group 3 = less affluent areas.

**Table 2:** Demographic data between in persons dying from non-cancer conditions diagnosed with or without depression the last year before death. Amount in each group (*n*) and row percentage in parenthesis.

Variables	Total *n* = 62,228	Depression *n* = 4391	No Depression *n* = 57,837	*p*-Value
Age, years, mean (sd)	76.9 (14.9)	71.6 (18.4)	77.3 (14.5)	<0.001
Age groups				<0.001
18–44 years, *n* (%)	2564	469 (18.3)	2095 (81.7)	
45–64 years, *n* (%)	7851	836 (10.7)	7015 (89.3)	
65–74 years, *n* (%)	11,565	702 (6.1)	10,863 (93.9)	
75–84 years, *n* (%)	18,264	1124 (6.2)	17,140 (93.8)	
85 years or more, *n* (%)	21,984	1260 (5.7)	20,724 (94.3)	
Sex				<0.001
Women, *n* (%)	27,473	2205 (8.0)	25,268 (92.0)	
Men *n* (%)	34,755	2186 (6.3)	32,569 (93.7)	
Diagnosis				
Parkinson’s disease	593	56 (9.4)	537 (90.6)	
COPD	4466	366 (8.2)	4100 (91.8)	
ALS	501	35 (7.0)	466 (93.0)	
Stroke	3859	258 (6.7)	3601 (93.3)	
Dementia	2172	133 (6.1)	2039 (93.9)	
Heart failure	8330	492 (5.9)	7838 (94.1)	
Mosaic-Socioeconomics				0.005
Group 1, *n* (%)	14,615	1118 (7.6)	13,497 (92.4)	
Group 2, *n* (%)	23,735	1619 (6.8)	22,116 (93.2)	
Group 3, *n* (%)	23,878	1654 (6.9)	22,224 (93.1)	
SPC				0.007
Yes	10,522	678 (6.4)	9844 (93.6)	
No	51,706	3713 (7.2)	47,993 (92.8)	
Place of death				<0.001
Acute hospitals, *n* (%)	24,482	1477 (6.0)	23,005 (94.0)	
Others, *n* (%)	37,746	2914 (7.7)	34,832 (92.3)	

Abbreviations: COPD = chronic obstructive pulmonary disease; ALS = amyotrophic lateral sclerosis. Mosaic group 1 = more affluent areas, Mosaic group 2 = middle-class areas, Mosaic group 3 = less affluent areas.

**Table 3:** Health care consumption and depression in the last year of life, last three months, and last month, respectively, of persons dying from non-cancer conditions.

Variable	Depression	No Depression	*p*-Value
Emergency room visits, *n* (%), last year of life	3931/4391 (89.5)	46,990/57,837 (81.3)	<0.001
Emergency room visits, *n* (%), last 3 months of life	3111/4391 (70.9)	40,097/57,837 (69.3)	0.03
Emergency room visits, *n* (%), last month of life	2389/4391 (54.4)	32,697/57,837 (56.5)	0.006
Visits in psychiatric services, *n* (%), last year of life	1816/4391 (41.4)	5078/57,837 (8.8)	<0.001
Visits in psychiatric services, *n* (%), last 3 months of life	1348/4391 (30.7)	3544/57,837 (6.1)	<0.001
Visits in psychiatric services, *n* (%), last month of life	1012/4391 (23.1)	2555/57,837 (4.4)	<0.001

**Table 4:** Uni- and multivariable regression models for prevalence of depression in the last year of life for persons dying in non-cancer conditions.

Variable	Univariable OR (95%CI)	*p*-Value	Multivariable aOR (95%CI) ^1^	*p*-Value
Age groups				
18–44 years	3.68 (3.28–4.13)	<0.0001	4.12 (3.66–4.63)	<0.0001
45–64 years	1.96 (1.79–2.15)	<0.0001	2.17 (1.98–2.39)	<0.0001
65–74 years	1.06 (0.97–1.17)	0.21	1.14 (1.04–1.26)	0.006
75–84 years	1.08 (0.99–1.17)	0.07	1.15 (1.05–1.24)	0.003
≥85 years	Ref.		Ref.	
Sex				
Women	1.30 (1.22–1.38)	<0.0001	1.46 (1.37–1.55)	<0.0001
Men	Ref.		Ref.	
Mosaic groups ^2^				
Group 1	1.11 (1.03–1.20)	0.008	1.19 (1.10–1.29)	<0.0001
Group 2	0.98 (0.92–1.01)	0.65	1.03 (0.96–1.11)	0.40
Group 3	Ref.		Ref.	

^1^ The c-statistic for the multiple regression model was 0.61. Hosmer and Lemeshow goodness-of-fit was 0.56. ^2^ Mosaic group 1 = the most affluent areas, Mosaic group 2 = middle-class areas, Mosaic group 3 = the least affluent areas.

**Table 5:** Sensitivity analysis: the multivariable model in Table 4 (above) was rerun in three separate models: (A) with the ICD-10 diagnosis F32 (Depressive episode) excluded; (B) with ICD-10 diagnosis F33 (Major depressive episode, recurrent) excluded, and (C) with all nursing home residents also included (nursing home residents are excluded from all other analyses).

	Model A: F32 Excluded, *n* = 1306		Model B: F33 Excluded, *n* = 3566		Model C: Nursing Homes Included, *n* = 7244	
Variable	Multivariable aOR (95%CI) ^1^	*p*-Value	Multivariable aOR (95%CI) ^2^	*p*-Value	Multivariable aOR (95%CI) ^3^	*p*-Value
Age groups						
18–44 years	8.70 (7.14–10.60)	<0.001	3.29 (2.89–3.75)	<0.001	4.21 (3.78–4.70)	<0.001
45–64 years	5.28 (4.47–6.25)	<0.001	1.73 (1.56–1.92)	<0.001	2.20 (2.02–2.38)	<0.001
65–74 years	2.00 (1.66–2.41)	<0.001	1.00 (0.90–1.11)	0.96	1.23 (1.14–1.33)	<0.001
75–84 years	1.52 (1.28–1.81)	<0.001	1.06 (0.96–1.16)	0.23	1.24 (1.17–1.32)	<0.001
≥85 years	Ref.		Ref.		Ref.	
Sex						
Women	1.59 (1.42–1.78)	<0.001	1.40 (1.31–1.46)	<0.001	1.35 (1.28–1.42)	<0.001
Men	Ref.		Ref.		Ref.	
Mosaic groups						
Group 1	1.26 (1.09–1.45)	0.002	1.18 (1.08–1.29)	<0.001	1.13 (1.06–1.20)	<0.001
Group 2	1.10 (0.97–1.25)	0.14	1.00 (0.92–1.09)	0.89	1.07 (1.01–1.13)	0.02
Group 3	Ref.		Ref.		Ref.	

^1^ When the diagnosis F32 (Depressive episode) was excluded, the c-statistic for the multiple regression model was 0.69, indicating a reasonable model. The Hosmer and Lemeshow goodness-of-fit was 0.56. ^2^ When the diagnosis F33 (Major depressive episode, recurrent) was excluded, the c-statistic for the multiple regression model was 0.59, implying a weak but statistically significant model. Hosmer and Lemeshow goodness-of-fit was 0.36. ^3^ When cases of depression in nursing home residents were also included, the c-statistic for the multiple regression model was 0.58, implying a weak but statistically significant model. The Hosmer and Lemeshow goodness-of-fit was 0.17.

## Data Availability

The raw data are available from the corresponding author upon reasonable request.

## Abbreviations

The following abbreviations are used in this manuscript:

ALS	Amyotrophic lateral sclerosis
aOR	Adjusted odds ratio
COPD	Chronic obstructive pulmonary disease
EOL	End-of-life
ICD-10	International classification of diseases 10th revision
Mosaic	A socioeconomic measure on area level
PD	Parkinson’s disease
SPC	Specialized palliative care
